# Supplementary material for: Reconfigurable emergent patterns in active chiral fluids
Source: Nat Commun. 2020 Sep 2;11:4401. doi: 10.1038/s41467-020-18209-x (PMC7468299; doi:10.1038/s41467-020-18209-x)
Supplement: Supplementary file 1 — Supplementary Information [file 41467_2020_18209_MOESM1_ESM.pdf]

Supplementary information for

Reconfigurable emergent patterns in active chiral fluids

Zhang et al.

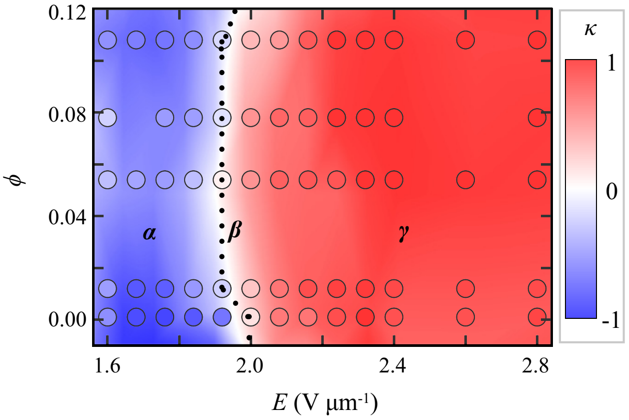

Supplementary Figure 1: **Trajectory curvature parameters, κ, as a function of the electric field strength obtained for a set of rollers densities.** Two distinctive modes of rolling correspond to mode α-'heads-out' (blue) and mode γ-'heads-in' (red) regimes of particles propulsion. The black dotted line shows the position of maximum values of persistence lengths at different area fractions of rollers shown in Fig.3.

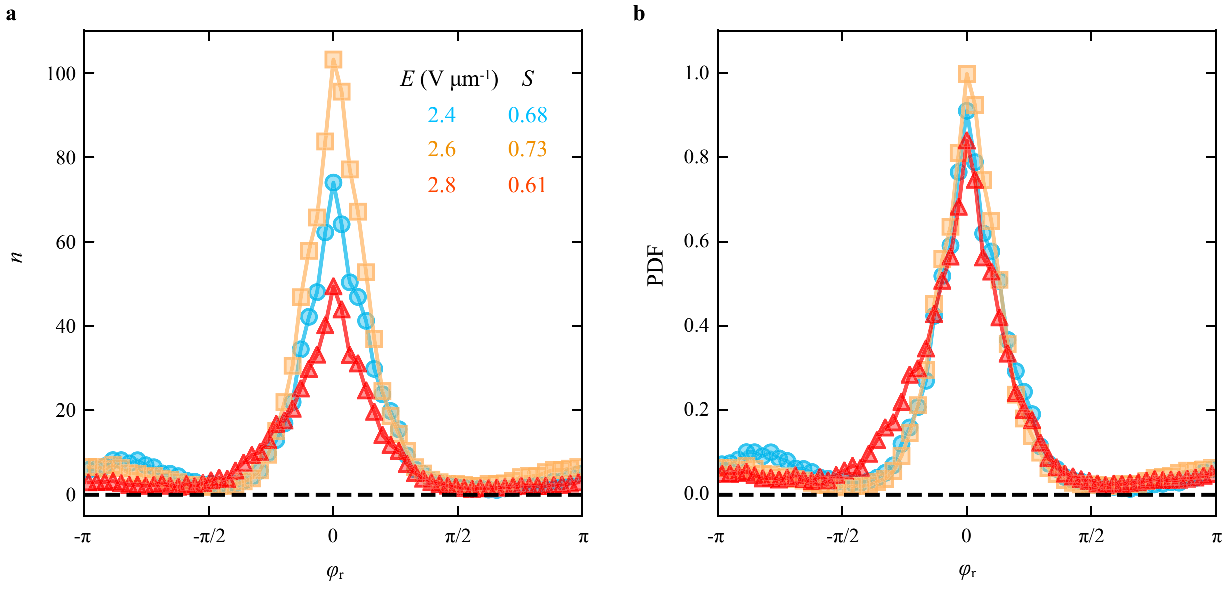

Supplementary Figure 2: **Polarization in the rotating flock phase.** **a**, The distributions of the orientation deviation angles, φ_{r}, with respect to the polarization direction in flock domains. The field strength, E, are 2.40 (blue), 2.60 (orange), and 2.80 (red) V µm^{-1}, respectively. $\phi = 0.254$. The values are averages over 2 largest domains at 50 different frames (total 100 domains) for each sample. Values of the nematic order parameters S for each distribution are also shown. **b**, Corresponding probability distribution functions (PDFs) of rollers' relative orientation φ_{r} in flock domains.

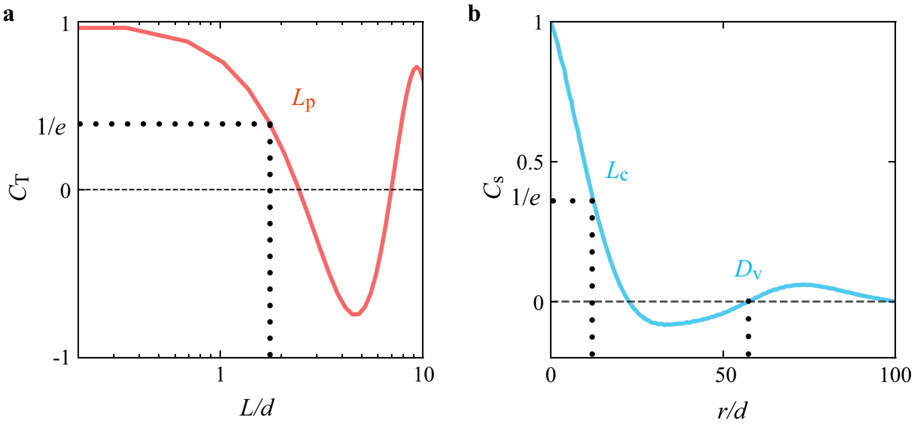

Supplementary Figure 3: **Definitions of characteristic lengths.** **a**, Persistence length, L_{p}, is defined as the distance particles travel where the velocity temporal correlation function C_{T} decays to $1/e$. **b**, Correlation lengths L_{c}, is defined as the distance where the spatial correlation function C_{s} decays to $1/e$. Characteristic vortex size, D_{v}, is defined as a second zero crossing in the spatial correlation curve C_{s}.

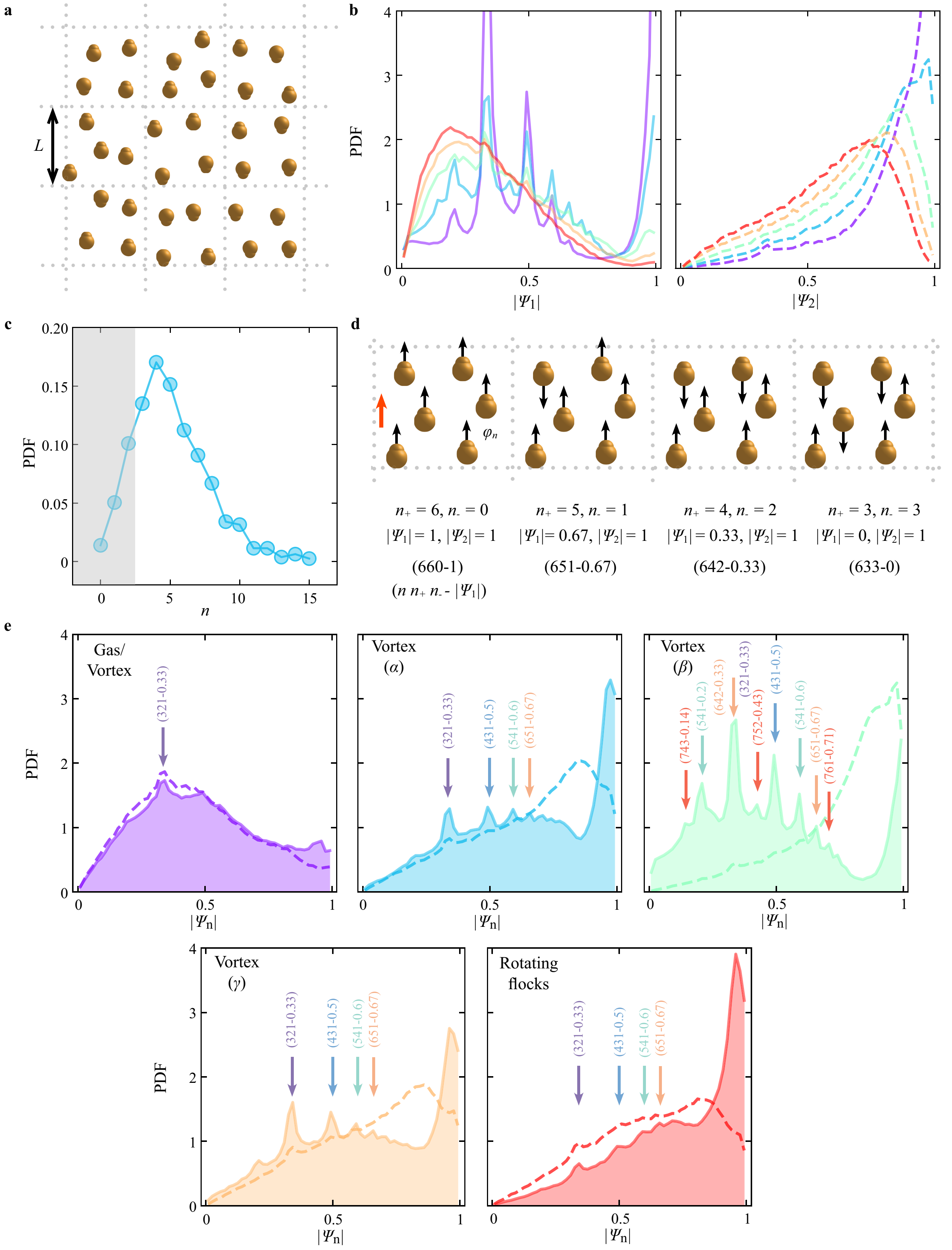

Supplementary Figure 4: **Local orientational order in the emergent patterns.** **a**, Particles are grouped into square boxes with sizes L. **b**, Probability distribution functions (PDFs) of the local orientational polar order parameters, $|\Psi_1|$ (left), and nematic order parameter, $|\Psi_2|$ (right) obtained for different box sizes L. L is $4d$ (purple), $6d$ (blue), $8d$ (green), $10d$ (orange), and $12d$ (red). $E = 1.92$ V µm^{-1} and $\phi = 0.108$. **c**, PDFs of number of particles, n, in each box. Only boxes with at least 3 particles are used to calculate order parameters. E and ϕ are the same as in **b**. **d**, Illustrations of possible configurations of particle directions ($n = 6$) with polar or nematic orders. n_+ and n_- are the number of particles pointing to same direction or opposite direction compared the reference direction (red arrow). Each configuration is named as (n n_+ n_- - $|\Psi_1|$). **e**, PDFs of $|\Psi_1|$ (solid lines) and $|\Psi_2|$ (dashed lines) for different collective patterns where the major satellites are assigned to a specific local configurations. The field strength, E, are 1.60, 1.76, 1.92, 2.08, and 2.40 V µm^{-1}, respectively. $\phi = 0.108$.

Supplementary Note 1

Polarization of the rotating flocks

In the rotating flock phase, multiple flock domains coexist. The domains are separated by domain boundaries (see Fig. 2d-e). The distributions of the orientation deviation angles with respect to the polarization direction of rollers forming the flocks and corresponding probability distribution functions (PDFs) are shown in Supplementary Figure 2a-b for different strength of the electric field. In each domain, particle orientations are well ordered as shown by the single narrow peak. The polarization within the rotating flocks can be further quantified by a scalar nematic order parameter,

$$S = \Big\langle \frac{3\,\cos^2(\varphi_{\rm r}) - 1}{2} \Big\rangle. \tag{1}$$

$\varphi_{\rm r}$ is the relative angle between the in-plain projection of a roller's orientation and the average direction of rollers in a flock; and $\langle ... \rangle$ indicates the average over rollers within the same flock.

The nematic order first increases with the field strength (as we move away from the phase boundary with vortices), peaks around 0.73 and decreases again as we move closer to the boundary with the spinner phase. A similar trend is also observed at a lower area fraction ϕ =0.108.

Supplementary Note 2

Local order of emergent patterns

As discussed in the main text, the orientational polar order parameter, Ψ_1, and the nematic order parameter, Ψ_2, are used to quantify the local polar and nematic order of particle directions in the system. Particles are grouped into square boxes with a size of L (Supplementary Figure 4a). A proper L is essential to detect the local order correctly. Too small grid boxes contain few particles while too large grid boxes contains too many particles and the local order will be averaged out. By comparing the probability distribution functions (PDFs) of $|\Psi_{\rm n}|$ for different L, $L = 6d$ is selected as an optimal size (Supplementary Figure 4b). With this size, most boxes contains 2-7 particles as shown Supplementary Figure 4c.

The satellite peaks in the probability density functions of the $|\Psi_{\rm n}|$ originate from the local disorder inside the grid boxes. Let's consider as an example a grid box where the particles can be oriented either parallel or antiparallel along a prescribed direction. In this case $|\Psi_2|$ is equal to 1 while $|\Psi_1|$ may take a range of values dependent of the number of particles with anti-parallel order. For the case of a grid box with six particles (shown in Supplementary Figure 4d), $|\Psi_1|$ can be 1 for (6,6,0-1) configuration, 0.67 for (6,5,1-0.67)configuration, 0.33 for (6,4,2-0.33) configuration or 0 for (6,3,3-0). Each configuration is encoded as (n, n_+, n_--$|\Psi_1|$), where n is a total number of particles in the box, and $\rm n_\pm$ is a number of particles with parallel(+)/antiparallel(-) orientations. These configurations contribute to corresponding satellite peaks in the probability distribution function of the $|\Psi_{\rm n}|$. See Supplementary Figure 4e where some of the major satellites are assigned to a specific local configurations. The positions and magnitudes of satellite peaks are the fingerprints of the local order(or disorder for that matter), the particle density variations, and different configurations of the particles local orientations.
