# Supplementary material for: Reconfigurable emergent patterns in active chiral fluids
Source: Nat Commun. 2020 Sep 2;11:4401. doi: 10.1038/s41467-020-18209-x (PMC7468299; doi:10.1038/s41467-020-18209-x)
Supplement: Supplementary file 3 — Description of Additional Supplementary Files [file 41467_2020_18209_MOESM3_ESM.pdf]

Description of Additional Supplementary Files

Supplementary Movie 1: Pear-shaped rollers in the vortex state (mode β). E = 1.92 V μm^{-1}. ϕ = 0.108. The movie is 21.5 times slower than the real time.

Supplementary Movie 2: β-vortex. E = 1.92 V μm^{-1}. ϕ = 0.108. The frame size is 0.25 mm by 0.24 mm. The movie is 21.5 times slower than the real time.

Supplementary Movie 3: Velocity and vorticity fields of pear-shaped rollers in the vortex state (mode β). E = 1.92 V μm^{-1}. ϕ = 0.108. The movie is 2.15 times slower than the real time.

Supplementary Movie 4: Pear-shaped rollers in the state of rotating flocks. E = 2.40 V μm^{-1}. ϕ = 0.254. The movie is 21.5 times slower than the real time.

Supplementary Movie 5: Rotating flock. E = 2.40 V μm^{-1}. ϕ = 0.254. The frame size is 0.25 mm by 0.25 mm. The movie is 21.5 times slower than the real time.

Supplementary Movie 6: Velocity and vorticity fields of pear-shaped rollers in the state of rotating flocks. E = 2.40 V μm^{-1}. ϕ = 0.254. The movie is 21.5 times slower than the real time.

Supplementary Movie 7: α-vortex. E = 1.76 V μm^{-1}. ϕ = 0.108. The frame size is 0.25 mm by 0.25 mm. The movie is 21.5 times slower than the real time.

Supplementary Movie 8: γ-vortex. E = 2.08 V μm^{-1}. ϕ = 0.108. The frame size is 0.25 mm by 0.25 mm. The movie is 21.5 times slower than the real time.

Supplementary Movie 9: Spinners. E = 2.80 V μm^{-1}. ϕ = 0.054. The frame size is 0.25 mm by 0.25 mm. The movie is 21.5 times slower than the real time.
